# Supplementary material for: Content-rich biological network constructed by mining PubMed abstracts
Source: BMC Bioinformatics. 2004 Oct 8;5:147. doi: 10.1186/1471-2105-5-147 (PMC528731; doi:10.1186/1471-2105-5-147)
Supplement: Additional File 2 — The original results of the above study (non-essential files are deleted to keep the file size under the limit set by BMC bioinformatics). [file 1471-2105-5-147-S2.bz2 › chilibotAdditionalFile2/dip10/668ID9122217E11068/html/TRAF3_LTBR.html]

 


 **TRAF3** and **LTBR** 
  
Found 22 abstracts in PubMed, retrieved 10.  
 

 What does Google say? 
 PDF only 
| .edu only 

---

**Interactive relationship** (e.g. stimulation, inhibition, etc)

**Non-interactive relationship** (e.g. studied together, co-existance, homology, etc.)

- Endogenous association of TRAF2,  **TRAF3** , cIAP1, and Smac with lymphotoxin beta receptor  [ **LTBR** ]  reveals a novel mechanism of apoptosis.  Ref: 12571250 J Biol Chem, 2003
- It takes part in the signal transduction of the TNF receptor 2 TNFR2, the lymphotoxin beta receptor  [ **LTBR** ]  LT betaR, CD40, CD30, and LMP1  [ **TRAF3** ] .  Ref: 9885224 Blood, 1999
